# C_2_H_5_OH and NO_2_ sensing properties of ZnO nanostructures: correlation between crystal size, defect level and sensing performance

**DOI:** 10.1039/c7ra13702h

**Published:** 2018-02-01

**Authors:** Chu Thi Quy, Nguyen Xuan Thai, Nguyen Duc Hoa, Dang Thi Thanh Le, Chu Manh Hung, Nguyen Van Duy, Nguyen Van Hieu

**Affiliations:** International Training Institute for Materials Science (ITIMS), Hanoi University of Science and Technology (HUST) No. 1, Dai Co Viet Road Hanoi Vietnam ndhoa@itims.edu.vn hieu@itims.edu.vn +84 24 38692963 +84 24 38680787

## Abstract

ZnO nanostructures can be synthesized using different techniques for gas sensor applications, but different synthesis methods produce different morphologies, specific surface areas, crystal sizes, and physical properties, which consequently influence the gas-sensing properties of materials. Many parameters such as morphology, specific surface areas, crystal sizes, and defect level can influence the gas-sensing properties of ZnO nanostructures. However, it is not clear which parameter dominates the gas-sensing performance. This study clarified the correlation between crystal size, defect level, and gas-sensing properties of ZnO nanostructures prepared from hydrozincite counterparts by means of field emission scanning electron microscopy, high resolution transmission electron microscopy, X-ray diffraction and photoluminescence spectra. Results showed that the average crystal size of the ZnO nanoparticles increased with thermal decomposition temperatures from 500 °C to 700 °C. However, the sample treated at 600 °C, which has the lowest visible-to-ultraviolet band intensity ratio showed the highest response to ethanol and NO_2_. These results suggested that defect level but not size is the main parameter dominating the sensor performance. The gas sensing mechanism was also elucidated on the basis of the correlation among decomposition temperatures, crystal size, defect level, and gas sensitivity.

## Abstract 2

ZnO nanostructures were synthesized for ethanol and nitrogen dioxide gas-sensing applications. Results pointed out that the defect levels dominating the gas-sensing performance but not the morphology, specific surface area or crystal size.

## Graphical abstract

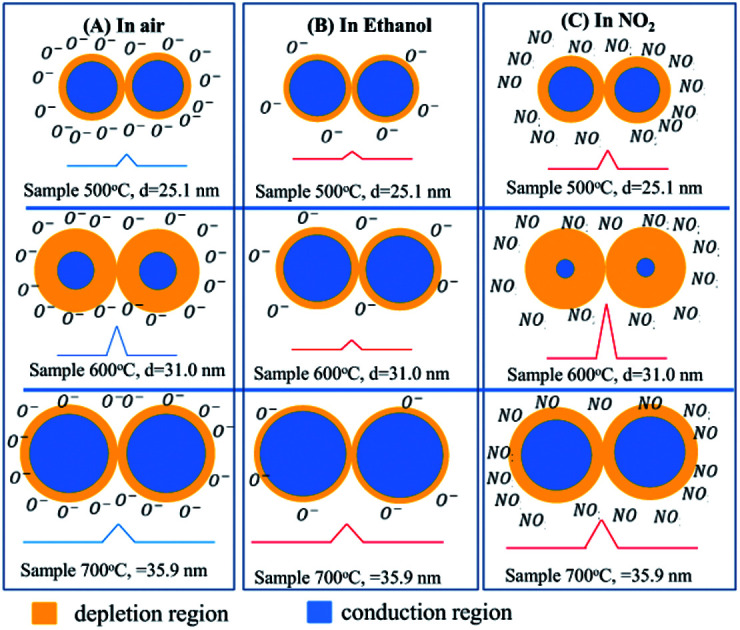

## 1. Introduction

Gas sensors have attracted increasing interest from researchers worldwide given their extensive applications in industrial emission control, household security, environmental monitoring, and disease diagnosis.^1–3^ Conductometric gas sensors^4^ based on metal oxides such as SnO_2_, WO_3_, Fe_2_O_3_, CuO, NiO, and ZnO have been technologically developed due to their small size, low cost, portability, reliability, robustness, and simple operation.^5–7^ Among gas-sensing materials, ZnO, a well-known n-type, direct wide-bandgap (3.37 eV) semiconductor with high electron mobility (210 cm^2^ V^−1^ s^−1^) and a large exciton binding energy (60 meV) has been the most attractive choice, thanks to its chemical and thermal stability, but still offering high response to toxic and combustible gases.^8–10^ ZnO nanostructures are advantages for gas sensor applications due to their small crystal size, large specific surface area, and turntable optical and electrical properties.^11,12^ Indeed, larger specific surface area provides more adsorption site for gaseous molecules to adsorb, while smaller crystal size (approximately to Debye length *λ*_D_) enables the total depletion, thus expected to show higher sensitivity.^13^ Because the Debye length, calculated by the equation *λ*_D_ = {2*k*_B_*T*/*e*^2^*N*_d_}^1/2^ is strongly dependent on the carrier density (*N*_d_) or the defect level. Therefore, by controlling the carrier density or the defect level, the effective Debye length of oxide can be adjusted to maximize the gas sensing response. For instance, by decreasing the carrier density can increase the Debye length which arts as reducing the effective crystal size of materials.

Literaturelly, many efforts have been dedicated for the enhancement of gas sensing performance of ZnO nanostructures by optimizing the synthesis processes or doping technique.^14^ For instance, Li *et al.*^15^ observed a strong visible emission peak centered at about 609 nm in ZnO nanorods (15 nm in diameter) as a result of large quantities of defects, thus enabled the high ethanol response by the total surface depletion. Different morphologies of ZnO nanostructures such as thin films,^16^ nanorods,^17^ nanowires,^18^ and nanoparticles^19^ have been also investigated for gas sensor applications. In our previous study,^20^ the gas sensing properties of ZnO nanorods, nanowires, and nanoparticles were systematically compared, where the nanoparticles exhibited higher ethanol sensitivity because of their smaller crystal size. In particularly, ZnO nanostructures can be prepared by different techniques such as chemical vapor deposition, sol–gel, hydrothermal, spray pyrolysis, precipitation, chemical bath deposition, successive ionic layer adsorption and reaction, and thermal deposition of correlative compounds.^21,22^ The last one appears to be the most effective and scalable method for synthesis of nanostructured ZnO with ability to control the crystal size, porosity, and physical characteristics by simply varying the decomposition temperatures.^22–24^ However, among the crystal size, specific surface area, and defect level, it is not clear which parameter mainly dominates the sensor response. As discussed above, defect level or carrier density is strongly influenced on the Debye length and thus the sensor response. It is believed that Zn interstitials and oxygen vacancies are common defects in ZnO, those are favored adsorption sites for gaseous molecules to adsorb.^25^ Experimentally evaluation the defect level can be done by comparing the optical properties of materials such as the PL spectra. For instance, Suranan *et al.*^26^ controlled the defect level by thermal annealing ZnO nanorods in oxidizing (O_2_) and reducing (Zn vapor) atmosphere; they observed a shift in defect level by scanning cathodoluminescence and pointed out that heat treated in oxidizing gas can enhance NH_3_ sensing performance. Zhang *et al.*^27^ studied the photoluminescence spectrum of ZnO nanostructures and found that the oxygen vacancy defects on the brush-shaped nanostructures was higher than that in nanowires, thus showed higher ethanol sensitivity. Al-Salman and colleagues reported that thin film of ZnO sputtering deposited on polyethylene telephthalate has a higher roughness and a higher visible emission intensity than those of thin film prepared on quartz substrates, and thus showing a higher sensitivity to hydrogen at room temperature.^28^ It is clearly that the defect level in ZnO nanostructures is very sensitive to the synthesis methods,^29^ where the thermal treatment is associated to structure and morphology transformations, thus affecting the size, shape, and physical properties of the resulting materials, which in turn determine the gas sensing response.^30^ However, the systematically study about the effect of heat treatment on the ZnO nanostructures prepared from hydrozincite nanoplates and their correction with their gas sensing properties are still very little known.^20,31^

In this study, we report the gas sensing properties of ZnO nanostructures obtained by thermally decomposition of plate-like hydrozincite at different temperatures ranging from 500 to 700 °C. Materials were characterized by FESEM, HRTEM, XRD, and PL spectroscopy, whereas their ethanol and NO_2_ sensing properties were tested at various working temperatures (250 to 400 °C). We aimed to understand the correlation between the crystal size, defect level and gas sensing properties of materials. Results pointed out that the increase in thermal decomposition temperature led to an increase in crystal size but the sample treated at 600 °C showed the highest response. The correlation between the material characteristics and gas sensing properties were clarified mainly by the defect level and crystal size.

## 2. Experimental

### 2.1. Materials synthesis

ZnO nanostructures were synthesized by thermal decomposition of precipitated hydrozincite at different temperatures. Sodium carbonate, zinc nitrate, ethanol (CH_3_CH_2_OH, purity 99.7%), purchased from Sigma-Aldrich company, and deionized-water were used as starting agents. Briefly, 50 mL of 1 M zinc nitrate was dropped to 10 mL of 1 M sodium carbonate at a flow rate of 10 mL min^−1^ while vigorous stirring at room temperature to precipitate hydrozincite. After that the precipitated hydrozincite was washed with distilled water and ethanol several times, and then collected *via* centrifugation (4000 rpm) before drying at 60 °C.^32^ Finally, ZnO nanostructures were produced by thermal decomposition of precipitated hydrozincite for about 2.5 h in air at temperatures ranging from 500 °C to 700 °C. Field emission scanning electron microscope (FESEM) images were recorded using a JEOL model JSM-7600F, while high resolution transmission electron microscope (HRTEM) images were measured using the JEOL JEM-2100F. The powder XRD was measured by a Bruker D8 advance using CuK-alpha, 0.154 nm irradiation. The room temperature photoluminescence (PL) emission spectra were recorded on a spectrophotometer (Nanolog, Horiba Jobin Yvon) using a 450 W xenon discharge lamp as an excitation source.^33^

### 2.2. Sensor fabrication

The sensors were fabricated by a thick film technique where the precipitated hydrozincite was re-dispersed in dimethylformamide to obtain a slurry and then spin coated on a thermally oxidized silicon substrate equipped with a pair of Pt electrode. The sensor chips were then cut into separate pieces for thermal treatment at different temperatures, noted as sample 500 °C, 600 °C, and 700 °C for annealing at 500 °C, 600 °C and 700 °C, respectively. The gas-sensing characteristics were measured at different temperatures using a Keithley Model 6220A as described elsewhere.^18^ Herein, the ethanol and NO_2_ gas sensing characteristics of the prepared sensors were studied for comparison. Standard gases with concentrations of 10 000 ppm (ethanol) and 100 ppm (NO_2_) were used in our experiments. To obtain a lower concentration, we mixed the standard gas with carrier gas (dry air) by using a series of mass flow controllers. By varying the flow rate ratio of standard and the carrier gases, we could obtain different concentration of test gases. During sensing measurement, the resistance of sensor was continuously recorded while the analytic gas was switched on/off. The sensor response was defined as *S* = *R*_gas_/*R*_a_ for NO_2_ and *S* = *R*_a_/*R*_gas_ for ethanol gas, where *R*_a_ and *R*_gas_ are resistances of sensor in dry air and in analytic gas, respectively. Detail about the experiment setup can be found in ref. 34.

## 3. Results and discussion

### 3.1. Materials characterizations

**Fig. 1:**
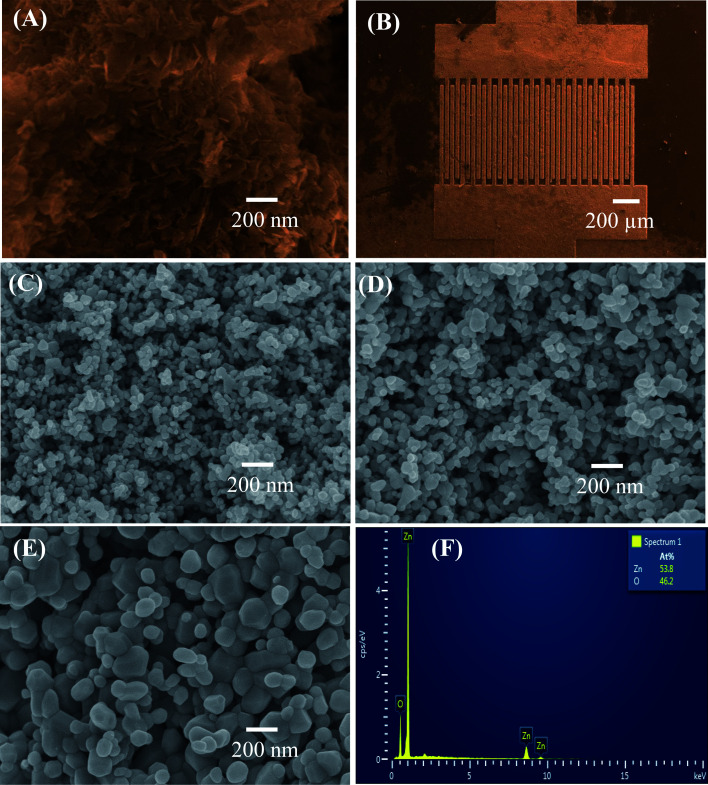
FE-SEM images of (A) hydrozincite, (B) sensor chip, and ZnO nanostructures annealed at different temperatures: (C) 500 °C, (D) 600 °C, (E) 700 °C; (F) EDS analysis of ZnO.

The morphology of the precipitated hydrozincite and ZnO nanostructures annealed at different temperatures was characterized *via* FESEM, as shown in Fig. 1. The hydrozincite [Fig. 1(A)] has a plate-like structure, where the average diameter and thickness of the plate-like particles were less than 100 and 10 nm, respectively. Fig. 1(B) shows a FESEM of a sensor chip, where the sensing materials were coated over and between the electrode fingers. The sensing layer is homogenous and thin enough, thus the electrodes can be seen easily. After thermal decomposition, the plate-like morphology of the hydrozincite transferred in to nearly spherical shapes of ZnO nanostructures to reduce the surface energy of the system. The ZnO nanoparticles are very homogenous with an average size of about 30 nm for sample 500 °C [Fig. 1(C)]. The size of ZnO nanostructures increase with increase of thermal decomposition temperatures, where the average particle sizes are approximately 50 and 100 nm for samples 600 °C, and 700 °C, respectively [Fig. 1(D) and (E)]. It is noticed that sample annealed at a higher temperature became less porous and inhomogeneous in particle size because of the grain growth where the small particles grew to become bigger to reduce the internal energy of system.^35^ The elemental analysis results of ZnO nanostructures *via* EDS are shown in Fig. 1(F). EDS confirmed the presence of Zn and O elements as main component of material. The atomic ratio between O and Zn [O]/[Zn] < 1 indicated the formation of non-stoichiometric ZnO_1−*x*_ (0 < *x* < 1), that representative for the n-type semiconducting of ZnO.^31^

**Fig. 2:**
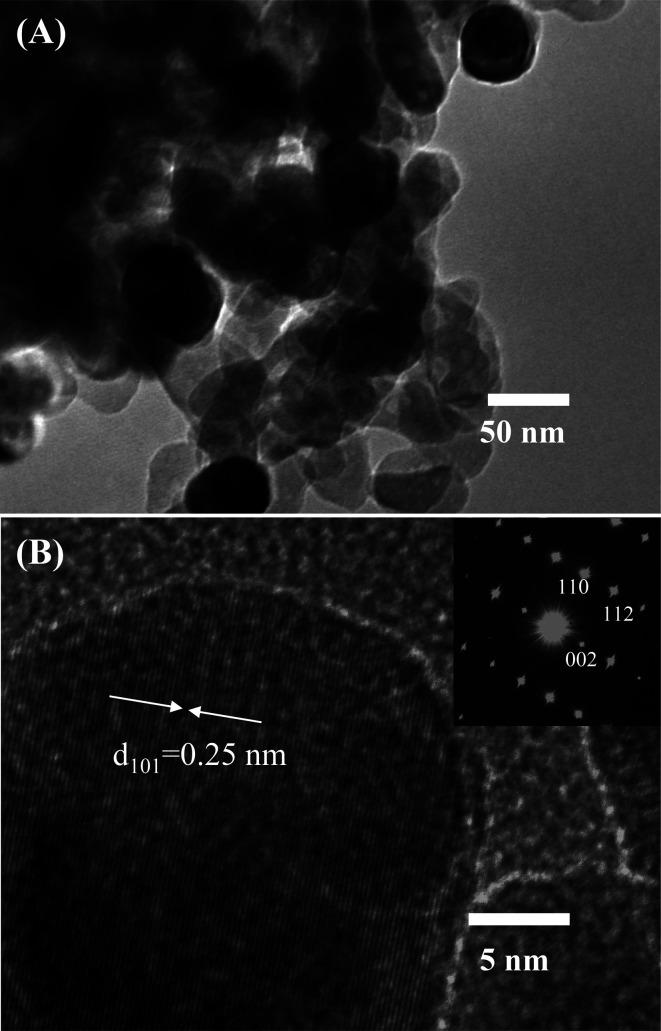
(A) TEM, and (B) HRTEM images of the synthesized ZnO nanostructures heat treated at 600 °C.

Further characterization about the morphology and crystallinity of the synthesized ZnO nanostructures were investigated by HRTEM images. As can be seen in Fig. 2(A), the TEM image of the ZnO nanostructures treated at 600 °C reveals an irregular share of the nanoparticles. The average size of the nanoparticles is about 50 nm, which is consistent with the observation by SEM images. HRTEM image of a ZnO nanoparticle shown in Fig. 2(B) demonstrates the single crystallinity of the material. The clear lattice fringes are observed in the HRTEM image with an interspacing of about 0.25 nm, which corresponds to the gap between two atomic planes of 101 (JCPDS 36-1451). Inset of Fig. 2(B) is a selective area electron diffraction, which confirms the single crystallinity of the ZnO nanoparticle.

**Fig. 3:**
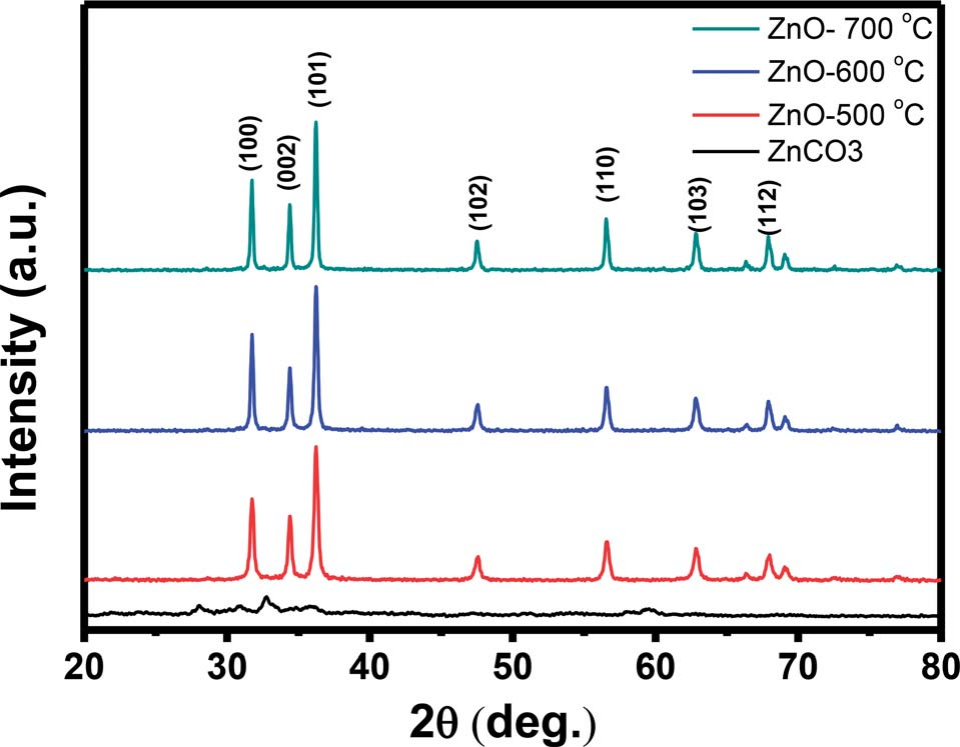
XRD patterns of ZnCO_3_ and ZnO annealed at 500 °C, 600 °C and 700 °C.

The crystal structure of the synthesized ZnO nanostructures was investigated *via* X-ray diffraction (XRD), and the results are shown in Fig. 3. The main diffraction peaks in the XRD patterns of the annealed samples were indexed to the wurtzite structure (hexagonal) of ZnO, with lattice constants of *a* = 3.249 Å and *c* = 5.206 Å (JCPDS 36-1451 card).^36^ No extra peaks related to any impurity were observed, confirming that the obtained nanostructures are single phase hexagonal ZnO. The diffraction peaks of the ZnO nanostructures annealed at higher temperatures (600 °C and 700 °C) are stronger and sharper than those of sample annealed at lower temperature (500 °C), confirming the grain growth with annealing temperatures. The average crystal sizes calculated by Scherrer formula using the (101) peaks were 25.1, 31.0, and 35.9 nm for samples treated at 500 °C, 600 °C and 700 °C, respectively. These values are quite small comparing to the values estimated from the FESEM images, possibly polycrystalline nature of the nanoparticles.

**Fig. 4:**
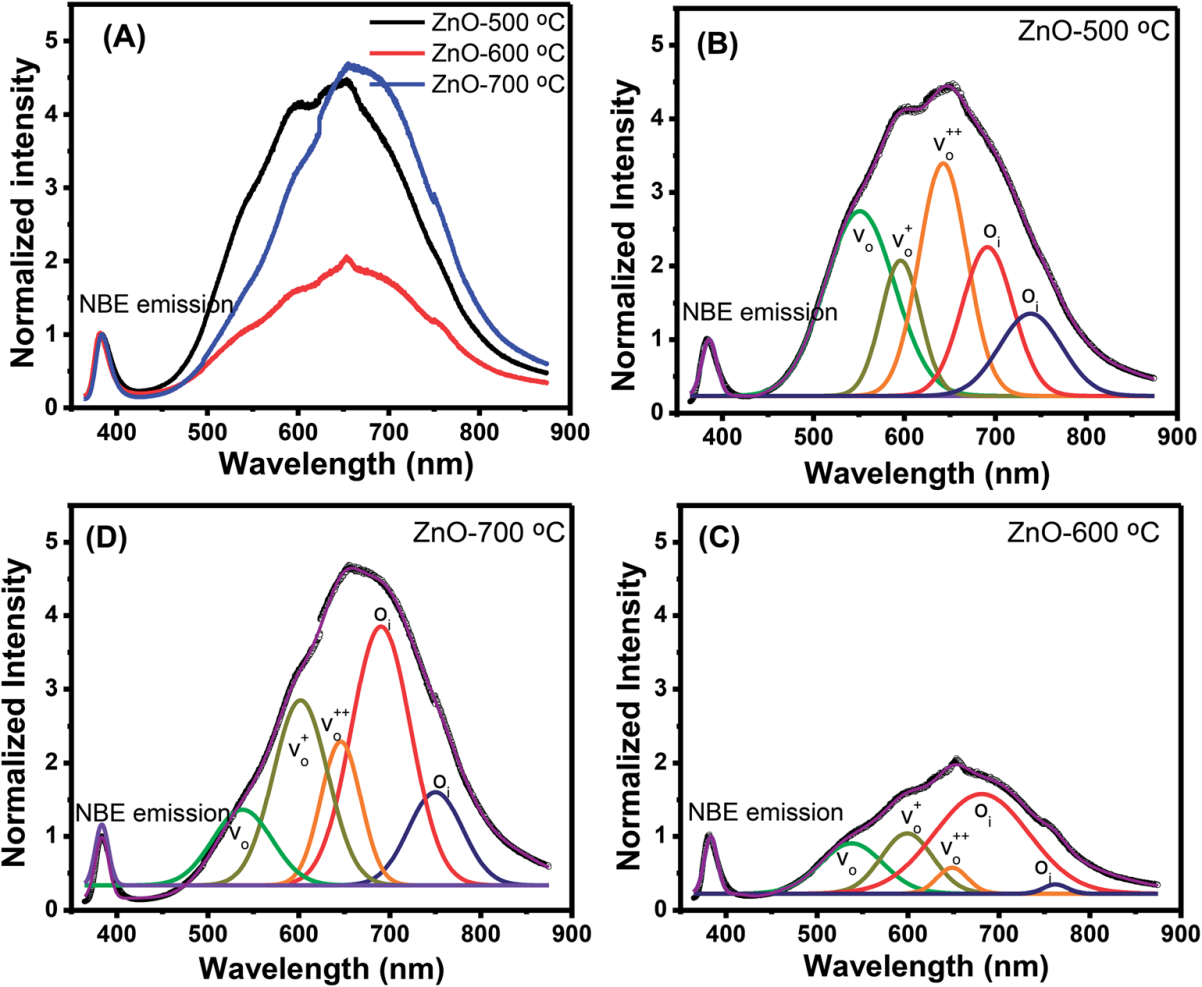
(A) PL spectra of ZnO nanostructures annealed at different temperatures; and deconvoluted PL spectra ZnO nanostructures annealed at (B) 500 °C; (C) 600 °C; (D) 700 °C.

Along with the XRD, photoluminescence (PL) is a suitable and nondestructive technique to determine the quality and the presence of impurities or defects in the materials.^37^ PL measurements were carried out at room temperature for all ZnO nanostructures treated at different temperatures. Fig. 4(A) shows the normalized PL spectra of the ZnO nanostructures annealed at different temperatures. In principle, the UV peak in the PL spectra is associated to the band-to-band emission while the visible emission originates from the defect levels.^38^ The PL spectra of ZnO nanostructures exhibited an UV emission band centered at about 383 nm (3.24 eV) and a broad emission peak at a visible region of about 650 nm (1.9 eV). The UV emission of sample 600 °C is centered at 380 nm (3.26 eV), while those of samples 500 °C and 700 °C peaked at 383 nm (3.24 eV). The band gap of quantum dots was believed larger than that of bulk counterpart, but herein they are smaller than the band gap of bulk ZnO (3.37 eV) suggesting the high defect level and substoichiometry of materials. The band gap narrowing was caused by the donor impurities because they create energy levels near the conduction band edge in the band gap. As a result, the effective band gap of highly donor doped semiconductor decreases. In the study by Kim *et al.*,^39^ where they studied the shift of bang gap in Ga doped ZnO thin films, and observed that the carrier concentration of ZnO decreases when the bandgap shift decreases. The band gap shift of samples 500 °C and 700 °C is 0.13 eV, whereas that value is 0.11 eV for sample 600 °C. Therefore, sample 600 °C has the lowest carrier density among others. In addition to the UV emission, the visible emission is also important because it related to the defect levels in ZnO, which includes zinc vacancies, interstitial zinc and lattice defects relating to oxygen and zinc.^33^ In this study, the relatively wide visible emission bands are observed ranging from 520 to 780 nm, and centered at 638, 654 and 668 nm, for samples 500 °C, 600 °C and 700 °C, respectively [Fig. 4(A)]. The red shift of visible band in ZnO nanostructures with increase of annealing temperatures from 500 to 700 °C can be explained by the grain growth in ZnO nanocrystals.^40^ As can be seen in the PL spectra of the ZnO nanostructures, the shape of the normalized PL spectra was not altered by the thermal treatment but the intensity and the width were. The sample 600 °C has the lowest visible band intensity. This is again confirmed the lowest defect level of carrier density of sample 600 °C. Wang *et al.*^41^ have shown an analogous effect for the ZnO thin films annealed for 2 h in air at 700 °C, 800 °C and 900 °C, where the visible emission peak intensity became stronger when the annealing temperature increased. Herein, sample 600 °C has the lowest visible emission intensity, and the largest band gap (3.26) among others. From these results it can be concluded that the thermal treatment has a great influence on the type and concentration of defects in ZnO nanostructures.^37^ To clarify which defects play a role on their gas sensing properties, we deconvoluted the visible peak (460–800 nm) of different samples, and the data are shown in Fig. 4(B–D). As can be seen, the visible peak in each sample could be deconvoluted into five peaks, those are neutral oxygen vacancies (*V*_O_), oxygen vacancies single charge 
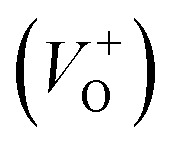
, oxygen vacancies double charge 
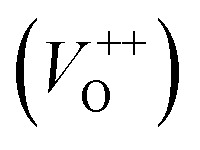
, and oxygen interstitials (O_i_).^42^ The position and percentages of each peak in different samples are summarized in Table 1. The total area of defect peak of the sample 600 °C is 310.08, while those values are 787.71 and 764.73 for the sample 500 °C and the sample 700 °C, respectively. This result again confirms the lowest defect in the sample 600 °C. As summarized in Table 1, the percentage of *V*_O_ decreased from 30.4% to 11.0% when the calcined temperature increased from 500 °C to 700 °C. The percentage of *V*_O_ is maximum in sample 500 °C with the value of 30.4%. However, the percentage of orange O_i_ is maximum in sample 600 °C with the value of approximately 56.5%, followed by the sample 700 (37.8%) and the sample 500 °C (18.1%). The results demonstrated that the *V*_O_ and O_i_ are strongly dependent on the calcined temperature. Note that the green emission was originated by the transition from conduction band to the deep levels oxygen vacancies including of *V*_O_ (520–570 nm), 
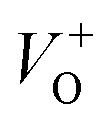
 (570–620 nm) and 
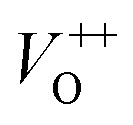
 (620–670 nm). The orange emission (670–720 nm) was attributed to the transitions from conduction band to O_i_ levels, while the red emission (720–780 nm) was original transition from Zn_i_ to O_i_.^33^ Therefore, moderate calcined temperature of 600 °C can generate more O_i_, but reduce 
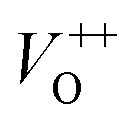
. Such those justification significantly influence to the gas sensing properties of the synthesized materials as discussed latter.

Summarized percentages of the PL spectra of ZnO nanostructures annealed at different temperatures de-convoluted by Gaussian distribution

**Table:** 

	*λ* (nm)	ZnO-500 °C	ZnO-600 °C	ZnO-700 °C
Total area of defect peak	460–800	787.71	310.08	764.73

**Table:** 

Portion in defect region (%)				
Origin	Peak (nm)	ZnO-500 °C	ZnO-600 °C	ZnO-700 °C
*V*_O_	520–570	30.4	18.8	11.0
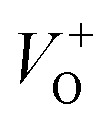	570–620	12.7	18.7	24.7
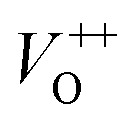	620–670	26.6	4.7	14.1
O_i_	670–720	18.1	56.5	37.8
O_i_	720–780	12.2	1.3	12.4

### 3.2. Gas-sensing properties

The voltage range from −5 V to +5 V was applied to all the samples in order to check the electrical contacts between the nanostructured ZnO and the Pt/Ti electrodes. The *I*–*V* plots (not shown) confirm a very good ohmic behavior, with negligible junction resistance. The resistance of the samples ranged from 7 MΩ to 350 MΩ in air at temperature from 400 down to 250 °C. The sensing properties of the ZnO mesoporous nanoparticles were tested by applying a voltage of 5 V between the electrodes and flowing different concentrations of analytics gas into the apparatus while measuring its current.

#### Ethanol sensing

**Fig. 5:**
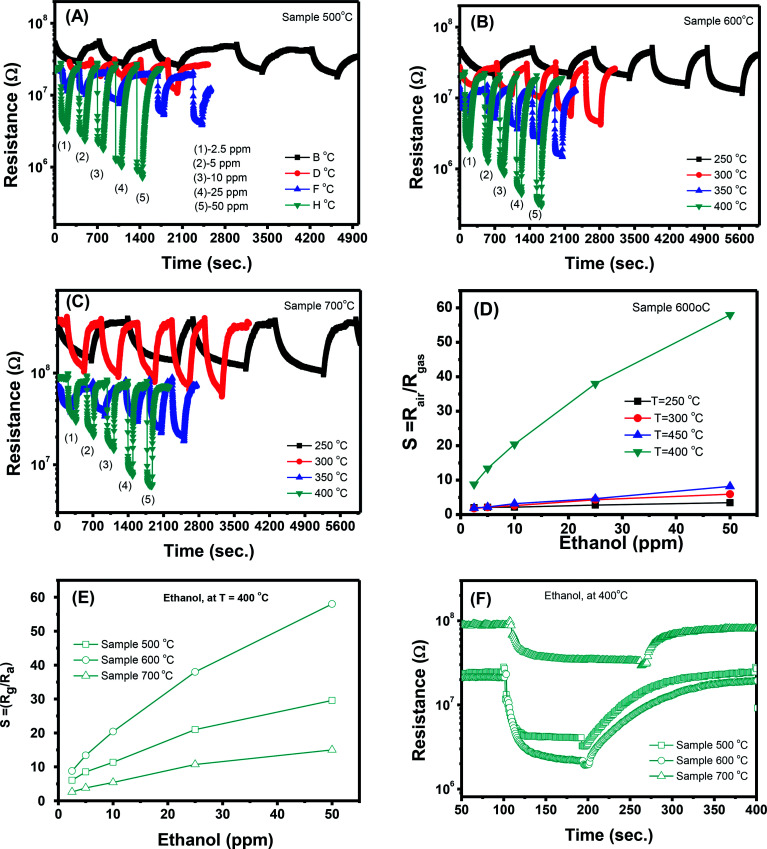
Transient resistance *versus* time of samples: (A) 500 °C, (B) 600 °C, (C) 700 °C; (D) response of sample 600 °C to different ethanol concentrations, (E and F) comparative results of different sensors at 250 °C.

The transient resistance *versus* time upon exposure to various ethanol concentrations (2.5, 5, 10, 25 and 50 ppm) measured at different working temperatures (250, 300, 350, and 400 °C) of samples 500 °C, 600 °C and 700 °C are shown in Fig. 5(A–C), respectively. At all measured temperatures the resistance of the ZnO sensors decreases steeply when ethanol gas is flown into the test chamber. However, the sensor resistance returns up to its previous value when the ethanol flow is interrupted and air is injected in the system. This trend confirms the n-type semiconductor behavior of ZnO sensors. When the ZnO nanostructures are exposed to air, oxygen molecules will be absorbed on the surface of the nanoparticles in the form of O^−^ and O^2−^. The surface layer of absorbed oxygen drains electrons from the ZnO nanoparticle core, increasing its resistance in air. When a reducing gas like ethanol is flown onto the sensors, its molecules interact with the pre-adsorbed oxygen layer releasing the electrons previously used in the chemical bonds back to the nanocrystals, and the sensor resistance decreases. Form the Fig. 5(A–C), it can roughly estimate that the response and recovery speeds of the sensors increase significantly with increase of working temperatures. For instance, at a measured concentration of 50 ppm, the response/recovery time of sample 500 °C is about 296/1078, 53/324, 11/256, 4/240 s, for temperatures of 250, 300, 350, and 400 °C, respectively. Details about the response and recovery time of different sensors are reported in Table 2.

**Table:** Response (Res.) and recovery (Rec.) time of different sensors

Conc., ppm	250 °C		300 °C		350 °C		400 °C	
	Res. time, s	Rec. time, s	Res. time, s	Rec. time, s	Res. time, s	Rec. time, s	Res. time, s	Rec. time, s
**Sample 500 °C**								
2.5	218	304	81	185	50	129	8	140
5	215	364	112	148	46	161	9	154
10	239	647	72	210	67	217	6	165
25	231	405	66	174	25	247	6	210
50	296	1078	53	324	11	>200	4	242

**Sample 600 °C**								
2.5	398	542	152	247	48	208	19	159
5	414	341	131	210	34	167	10	181
10	322	265	93	175	16	149	7	205
25	212	233	43	209	11	142	11	262
50	166	313	64	207	5	166	6	329

**Sample 700 °C**								
2.5	423	269	164	261	149	300	30	182
5	421	241	145	259	117	242	15	218
10	401	256	172	197	123	144	8	196
25	374	441	141	223	94	117	7	191
50	393	361	135	251	73	197	6	161

In addition to the response and recovery speeds, the sensor resistance also dynamically changes when different ethanol concentrations are injected into the system. The response magnitude improves with increasing gas concentration at all working temperature. A more quantitative analysis for sample 600 °C can be extracted from the dynamic resistance plots, as reported in Fig. 5(D). It is clearly that the sensor response increases with increasing ethanol concentrations at all working temperatures. However, that the measured temperature of 400 °C, the sensor has the highest response values which are few times higher than the others and without evidence of saturation in the range of investigated ethanol concentrations. Herein, working temperature higher than 400 °C was not measured because of the limitation of the sensing system. In addition, higher working temperature means requiring higher power consumption, and also lead to decline of the stability due to the grain growth. A comparative about the response of different sensors measured at 400 °C is shown in Fig. 5(E). It is very interesting that the sample 600 °C, with the lowest area (intensity) of the visible emission peak shows the highest response and its response values increases quickly with increasing ethanol concentration, from 8.8 to 58 when ethanol concentration increasing from 2.5 to 50 ppm. This could be attributed to the correlation between the increase of crystal size of ZnO nanostructures with increasing treated temperatures and the defect levels in the materials.^43^ With increase of annealing temperatures, the crystal size increases but the defect level or carrier density decreases. As a results, the effective Debye length increase, and thus the ZnO nanocrystals is total depleted and maximize the sensor response.^15^

Ethanol sensing mechanism can be explained as follow

1 C_2_H_5_OH + 3O^−^_2_ ↔ 2CO_2_ + 3H_2_O + 3e^−^

2 C_2_H_5_OH + 6O^−^ ↔ 2CO_2_ + 3H_2_O + 6e^−^

3 C_2_H_5_OH + 6O^2−^ ↔ 2CO_2_ + 3H_2_O + 12e^−^

The interaction between ethanol molecules and pre-adsorbed oxygen releases electrons back to the n-type ZnO crystals and reduces the space–charge layer, resulting in decreased sensor resistance.^32^ As mentioned above, the Debye length is dependent on the carrier density, thus samples annealed at different temperatures have different Debye length. It is believed that the sample 600 °C have a compatible value of crystal size and Debye length, thus enable the total depletion, and maximize the sensor response.

#### NO_2_ sensing characteristics

**Fig. 6:**
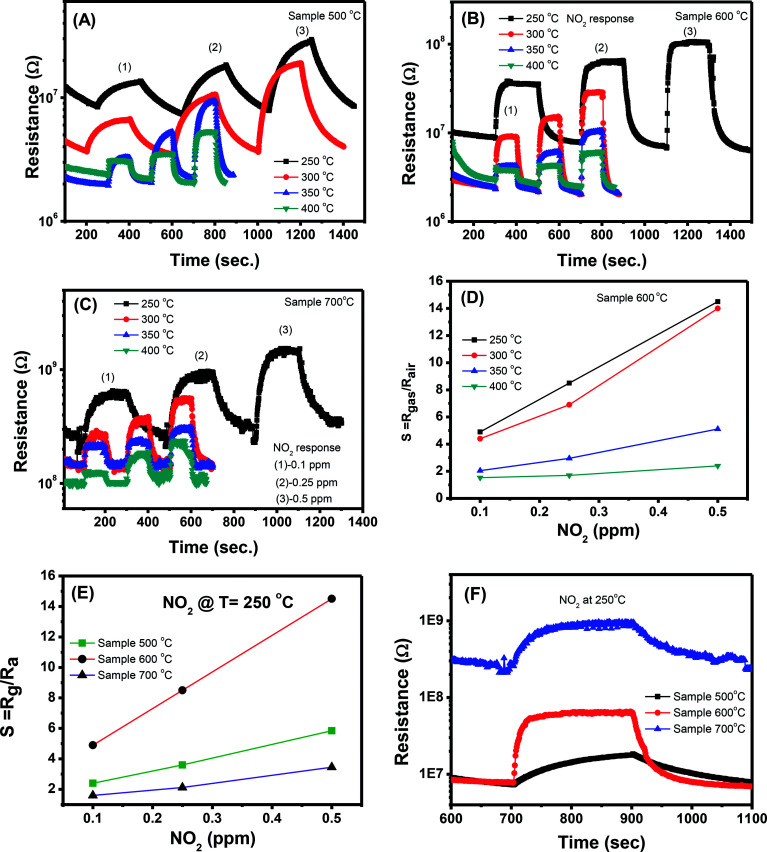
Transient resistance *versus* time of samples: (A) 500 °C, (B) 600 °C, (C) 700 °C; (D) response of sample 600 °C to different NO_2_ concentrations, (E and F) comparative results of different sensors at 250 °C.

The transient resistance *versus* time of a sample 500 °C as a function of NO_2_ concentration (0.1, 0.25 and 0.5 ppm) at different working temperatures (250, 300, 350, and 400 °C) is shown in Fig. 6(A). At all measured temperatures, the sensor resistance increases steeply when NO_2_ gas is flown into the system. The sensor resistance removes to its initial value when the NO_2_ flow is interrupted and dry air is injected in the system. This trend confirms the n-type semiconductor behavior of ZnO because NO_2_ molecule can capture electrons and adsorb on the surface of ZnO in the forms of NO_2_^−^, NO_3_^−^ or NO^−^ and thus increases the sensor resistance. NO_2_ has a higher electron affinity (2.28 eV) in comparison with oxygen (0.43 eV), thus when adsorbed on the surface of ZnO it can capture more electrons and increase the sensor resistance.^44^ Adsorption of NO_2_ on the surface of ZnO is complex but it can be expressed by the following equation.^44,45^

4 NO_2(gas)_ + e^−^ = NO^−^_2(ads)_

5 NO_2(gas)_ + O^−^ = NO^−^_3(ads)_

6 NO_2(gas)_ + O^−^ = 2NO^−^_(ads)_

7 NO^−^_2(ads)_ + O^−^ = NO_2(gas)_ + O^2−^_ads_

However, with increase of working temperature, the response and recovery speeds are increase as a result of the thermal acceleration for reaction and desorption of gaseous molecules. Fig. 6(B) and (C) show the resistance changes to various nitrogen dioxide concentrations at different working temperatures of the samples 600 °C and 700 °C, respectively. As can be seen, all the samples show a similar trend in response where the sensor resistance increases with introduce of NO_2_ gas. A more quantitative analysis can be extracted from the dynamic resistance plots, as shown in Fig. 6(D) for the sample 600 °C. The sensor response increases with increasing NO_2_ concentration at all measured temperatures. The response values also increase with decrease of working temperatures from 400 to 250 °C. This mean that the sensor response is higher at a lower working temperature. However, we did not measure the sensor response at lower 250 °C because it required long time for response and recovery. Fig. 6(E) compares the response of different sensors measured at 250 °C. It is clearly that sample 600 °C shows the highest response among others. This result is consistent with the ethanol response which confirms that thermal treatment strongly influences on the gas sensing properties of ZnO nanostructures. Among the factors, we can conclude that the defect level or carrier density dominates the ethanol and NO_2_ sensitivity of ZnO nanostructures.

**Fig. 7:**
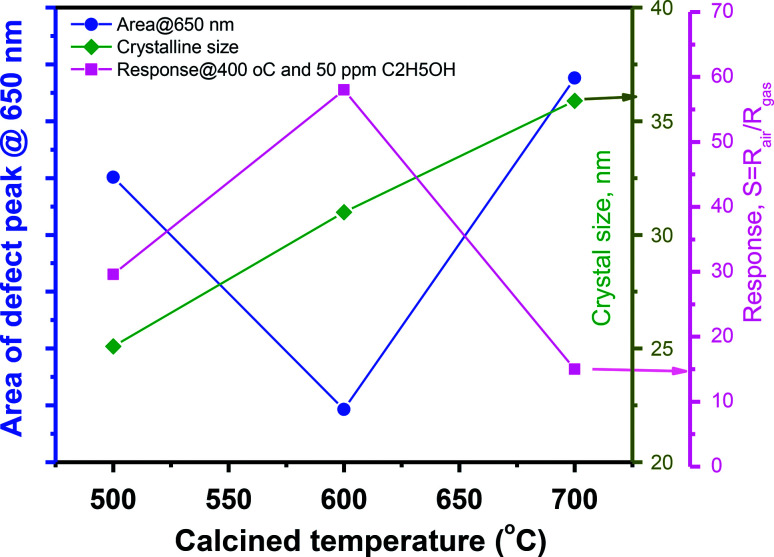
The correlation of sensor response, crystal size and point defects (area of visible peaks) as a function of calcined temperatures.

Correlation of sensor response, point defects (defined as area of visible peaks) and crystal size as a function of calcined temperatures is shown in Fig. 7. The crystal size increased from 25.1 nm to 35.9 nm with increase of calcined temperature from 500 °C to 700 °C. It was expected that smaller crystal size will result to higher defects but it was not in our study, where the sample calcined at 600 °C (with a medium crystal size) has the smallest defects and showing the highest response. As summarized in the Table 1, the main defect in the sample 600 °C is orange O_i_ of approximately 56.5%. The O_i_ is acceptor doping in the n-type ZnO, thus it reduces the carrier density (*N*_d_) and increases the Debye length because this value is inversely proportional to the square root of *N*_d_. As a result, the large amount of O_i_ can increase the sensor response.

**Fig. 8:**
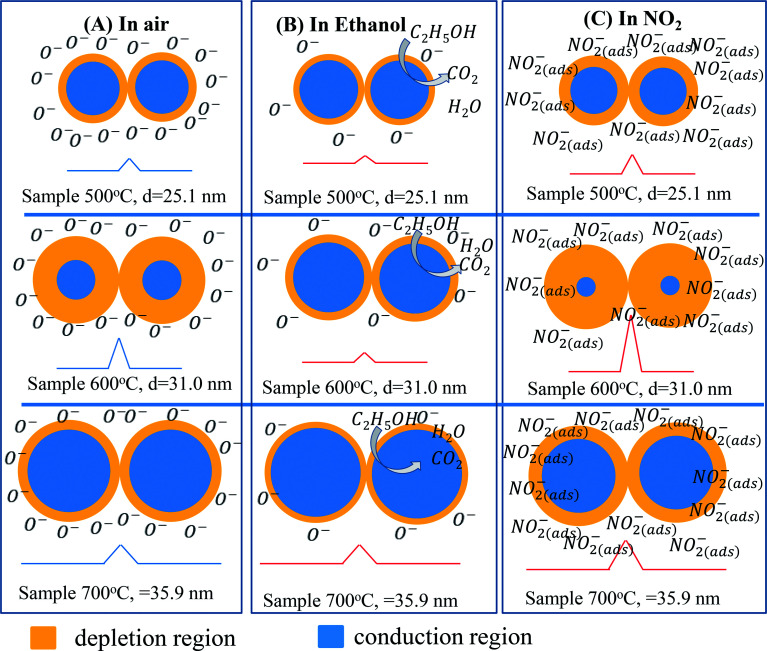
Schematic diagram of ZnO intergranular with depletion region and barrier height in air (A); ethanol (B), and NO_2_ (C) of nanostructure ZnO with different sizes.

Gas sensing mechanism of the ZnO nanostructures with various defect levels and different crystal sizes can be explained by the diagram shown in Fig. 8. Because the sensor 600 °C has low defect level thus its Debye length is strongly dependent on the ambient environment. In air, the barrier height in this sample is relatively higher than that in other samples. When exposure to analytic gas, the barrier height in this sample significantly decreases (in ethanol) or increases (in NO_2_), thus showing the higher response.^44^

## 4. Conclusions

ZnO nanostructures were produced through a simple and inexpensive wet-chemical method followed by thermal treatment at various temperatures ranging from 500 °C to 700 °C for gas sensor applications. ZnO nanostructures with average crystal sizes ranging from 25.1 to 35.9 nm were obtained simply by varying the thermal treatment temperatures. Material characterization demonstrated that the thermal treatment significantly influenced on the crystal size and optical properties, which in turn controlled the gas sensing performance. The synthesized ZnO nanostructures showed good response to ethanol and NO_2_ at very low concentrations, but the sample treated at 600 °C exhibited the highest response values both to ethanol and NO_2_. We also demonstrated that the defect level dominated the sensor response but not the crystal size. Lower defect level exhibited higher ethanol and NO_2_ response. Our results suggested an effective method to maximize the gas sensitivity of ZnO nanostructures for developing a new class super sensitive sensor to ethanol and NO_2_ at low concentrations.

## Conflicts of interest

There are no conflicts to declare.

## Supplementary Material
